# Age-Dependent Changes in Resting Energy Expenditure (REE): Insights from Detailed Body Composition Analysis in Normal and Overweight Healthy Caucasians

**DOI:** 10.3390/nu8060322

**Published:** 2016-06-01

**Authors:** Corinna Geisler, Wiebke Braun, Maryam Pourhassan, Lisa Schweitzer, Claus-Christian Glüer, Anja Bosy-Westphal, Manfred J. Müller

**Affiliations:** 1 Institute of Human Nutrition and Food Science, Christian-Albrechts-Universität zu Kiel, Düsternbrooker Weg 17-19, D-24105 Kiel, Germany; cgeisler@nutrfoodsc.uni-kiel.de (C.G.); wbraun@nutrfoodsc.uni-kiel.de (W.B.); mpourhassan@nutrfoodsc.uni-kiel.de (M.P.); lschweitzer@nutrfoodsc.uni-kiel.de (L.S.); 2 Clinic for Diagnostic Radiology, Section Biomedical Imaging, Molecular Imaging North Competence Center (MOIN CC), Am Botanischen Garten 14, D-24118 Kiel, Germany; glueer@rad.uni-kiel.de; 3 Institute of Nutritional Medicine, Universität Hohenheim, Fruwirthstr. 12, D-70599 Stuttgart, Germany; Anja.Bosy-Westphal@uni-hohenheim.de

**Keywords:** age, body composition, resting energy expenditure, MRI, metabolic risk

## Abstract

Age-related changes in organ and tissue masses may add to changes in the relationship between resting energy expenditure (REE) and fat free mass (FFM) in normal and overweight healthy Caucasians. Secondary analysis using cross-sectional data of 714 healthy normal and overweight Caucasian subjects (age 18–83 years) with comprehensive information on FFM, organ and tissue masses (as assessed by magnetic resonance imaging (MRI)), body density (as assessed by Air Displacement Plethysmography (ADP)) and hydration (as assessed by deuterium dilution (D_2_O)) and REE (as assessed by indirect calorimetry). High metabolic rate organs (HMR) summarized brain, heart, liver and kidney masses. Ratios of HMR organs and muscle mass (MM) in relation to FFM were considered. REE was calculated (REEc) using organ and tissue masses times their specific metabolic rates. REE, FFM, specific metabolic rates, the REE-FFM relationship, HOMA, CRP, and thyroid hormone levels change with age. The age-related decrease in FFM explained 59.7% of decreases in REE. Mean residuals of the REE-FFM association were positive in young adults but became negative in older subjects. When compared to young adults, proportions of MM to FFM decreased with age, whereas contributions of liver and heart did not differ between age groups. HOMA, TSH and inflammation (plasma CRP-levels) explained 4.2%, 2.0% and 1.4% of the variance in the REE-FFM residuals, but age and plasma T3-levels had no effects. HMR to FFM and MM to FFM ratios together added 11.8% on to the variance of REE-FFM residuals. Differences between REE and REEc increased with age, suggesting age-related changes in specific metabolic rates of organs and tissues. This bias was partly explained by plasmaT3-levels. Age-related changes in REE are explained by (i) decreases in fat free mass; (ii) a decrease in the contributions of organ and muscle masses to FFM; and (iii) decreases in specific organ and tissue metabolic rates. Age-dependent changes in the REE-FFMassociation are explained by composition of FFM, inflammation and thyroid hormones.

## 1. Introduction

Resting energy expenditure (REE) decreases from young to old age by 1% to 2% per decade [1]. This is partly explained by age-related decreases in fat free mass (FFM) [2]. FFM accounts for 50%–70% of the variance in REE [3,4,5]. Metabolically, FFM is heterogeneous including high (*i.e.*, heart, liver, kidneys and brain) and low metabolic rate organs and tissues (*i.e.*, muscle mass and skeletal bone) [6]. In young adults, brain, liver, heart and kidney masses add up to approximately 12% of FFM but account for 60% of REE [3,7,8,9,10,11]. By contrast, muscle mass comprises more than 50% of FFM and accounts for up to 25% of REE only [5]. We have shown previously that REE and FFM change with age with gender-specific differences in the onset and magnitude of the age-related changes in metabolically active body components and REE [12]. Decreases in REE and body composition started between 30 and 45 years with decreases in REE adjusted for skeletal muscle and organ mass and adipose tissue by −145 kJ/day/decade and −604.8 kJ/day/decade after the age of 35.2 and 34.3 years in women and men, respectively [12]. There was first evidence that specific metabolic rates of major organs and tissues also decrease with age, and age-specific prediction algorithms have been published by Wang *et al.* [11,13]. Thus, the present evidence suggests that age-related decreases in REE are explained by decreases in both (i) FFM and (ii) the specific metabolic rate of organs and tissues. However, age-associated changes in the REE-FFM relationship still remain to be characterized.

The nature and the impact of age-related changes in organ and tissue masses together with changes in REE have not been widely examined. Two longitudinal studies, the Baltimore Longitudinal Study (BLSA) and Health Aging and Body Composition Study (Health ABC), indicated that a high resting metabolic rate at an older age was a risk factor of mortality [14] as well as for multi-morbidity in men [15,16]. The data of another longitudinal study, the German GISELA study [17] showed that REE decreases by 11.2 kJ/day and 34.1 kJ/day per year in women and men, respectively. However, in these studies, detailed composition of FFM has not been assessed.

In this cross-sectional study, we investigated a greater population of young and old adults with normal and overweight (BMI < 30 kg/m^2^) in order to avoid an obesity bias with consideration that a recommendation of a normal BMI for older adults is higher (BMI 24.0 to 29.0 kg/m^2^) than for younger adults. The aims of the study were to describe the age-related differences and the impact of the REE-FFM association, taking into account detailed body composition data as obtained by whole body MRI.

## 2. Materials and Methods

Data from the “Reference Center for Body Composition” (Institute of Human Nutrition and Food Science of the Christian-Albrechts University Kiel, Germany) were used in this secondary data analysis (Table 1). Subjects had participated in different studies on body composition and metabolism [7,10,11,18,19,20]. The total number of subjects with BMI < 30 kg/m^2^ was 714 (346 women and 368 men) with a median age of 41.0 years (18–83 years) and a median BMI of 24.6 kg/m^2^ (16.8–29.9 kg/m^2^). Data of tissue and organ masses assessed by whole body magnetic resonance imaging (MRI) were available in a subgroup of 369 healthy Caucasians (168 women and 201 men).

Studies were approved by the ethical committee of the department of medicine (Christian-Albrechts University Kiel; last approved version A100/13A; 2014) and informed written consent to participate in the study was obtained from each subject. All studies were conducted according to the guidelines laid down in the “Declaration of Helsinki”.

Body height was measured to the nearest 0.5 cm with subjects wearing no shoes (secastadiometer; Hamburg, Germany). Weight was assessed to the nearest 0.01 kg with an electronic scale (Tanita, Tokyo, Japan).

Body composition was assessed by Air Displacement Plethysmography (ADP). ADP was performed by the BOD POD^®^ device (Cosmeds.r.l., Rome, Italy). Participants wore tight-fitting underwear and a swim cap. Two repeated measurements of body volume were performed, averaged and corrected for predicted body surface area and thoracic gas volume using BOD POD^®^ software (version 4.5.0). Percentage fat mass (FM_ADP_) was calculated from body density using the equation by Siri *et al.* [21]. Fat free mass (FFM_ADP_) was calculated as body weight minus FM_ADP_. Total body water was assessed by dilution method as previously described in detail [22] and used to calculate the hydration of FFM_ADP_.

In a subgroup of 369 subjects with BMI < 30 kg/m^2^ muscle mass (MM), total, subcutaneous and visceral adipose tissue and organ masses were measured using whole body multislice MRI. Scans were obtained with a 1.5T scanner (Magnetom Vision Siemens, Erlangen, Germany) as previouslydescribed [23]. Areas and volumes of MM, adipose tissue and volumes of 5 internal organs (brain, heart, liver, spleen and kidney) were manually analyzed using the SliceOmatic software (version 4.3; Tomovision, Montreal, QC, Canada) as described earlier [8]. Intra-observer coefficient of variation (CV) was 1.8% for total SM, 1.8% for brain, 0.07% for liver, 1.7% for heart and 1.0% for kidney.

DXA whole body measurement was performed (QDR4500A, Hologic Inc., Bedford, MA, USA). Subjects laid in supine position during the 10 min scan. Manufactures software (version V8.26a:3, Hologic Inc., Bedford, MA, USA) was used for analysis of bone mineral content (BMC_DXA_). Skeletal bone massDXA was calculated as BMC_DXA_ × 1.85 and included in calculations of resting energy expenditure [24].

Resting energy expenditure (REE) was measured by indirect calorimetry (REE) with an open-circuit ventilated-hood system (Vmax Spectra 29n, SensorMedics BV, Viasys Healthcare, Bilthoven, The Netherlands; software V-max version 12-1A). REE was measured in the early morning after an overnight fast, and a detailed description of the measurement was reported elsewhere [7,25]. The CV for repeated measurements of REE was 5.0% [26]. In addition to measured REE, REE was calculated (REEc) based on organ and tissue masses times specific tissue metabolic rates reported by Elia *et al.* [27] and Wang *et al.* [13], who have published age-corrected values. For skeletal bone massDXA, a specific metabolic rate of 9.63 kJ/(kg·day) was assumed [28]. The REE equations were as follows:

REEc Equation published by Elia *et al.* [27]:

REEc [kJ/day] = (1008 (kJ) × brain mass (kg)) + (840 (kJ) × liver mass (kg)) + (1848 (kJ) × heart mass (kg)) + (1848 (kJ) × kidney mass (kg)) + (55 (kJ) × skeletal muscle mass (kg)) + (19 (kJ) × adipose tissue (kg)) + (9.63 (kJ) × skeletal bone massDXA (kg)) + (50 (kJ) × residual mass (kg))
(1)

Age-related REEc equations published by Wang *et al.* [13]:

21–30 years

REEc [kJ/day] = (1016 (kJ)×brain mass (kg)) + (848 (kJ)×liver mass (kg)) + (1860 (kJ) × heart mass (kg)) + (1860 (kJ) × kidney mass (kg)) + (55 (kJ) × skeletal muscle mass (kg)) + (19 (kJ) × adipose tissue (kg)) + (9.63 (kJ) × skeletal bone massDXA (kg)) + (51 (kJ) × residual mass (kg))
(2)

31–50 years

REEc [kJ/day] = (1003 (kJ)×brain mass (kg))+(835 (kJ)×liver mass (kg)) + (1839 (kJ) × heart mass (kg)) + (1839 (kJ) × kidney mass (kg)) + (54 (kJ) × skeletal muscle mass (kg)) + (19 (kJ) × adipose tissue (kg)) + (9.63 (kJ) × skeletal bone massDXA (kg)) + (50 (kJ) ×residual mass (kg))
(3)

51–73 years

REEc [kJ/day] = (978 (kJ) × brain mass (kg)) + (814 (kJ) × liver mass (kg)) + (1789 (kJ) × heart mass (kg)) + (1789 (kJ) × kidney mass (kg)) + (53 (kJ) × skeletal muscle mass(kg)) + (18 (kJ) × adipose tissue (kg)) + (9.63 (kJ) × skeletal bone massDXA (kg)) + (48 (kJ)×residual mass (kg))
(4)

Blood samples were taken after an overnight fast and insulin, glucose, thyroid hormones, and C-reactive proteins were analyzed as previously described [29,30].

Statistical analysis was performed using SPSS statistical software (SPSS 22.0, Inc., Chicago, IL, USA). All data are given as median+interquartil range (IQR). Differences between women and men were tested by Mann-Whitney U-test and between age groups using the Kruskal-Wallis-test. To assess differences of age-related changes in organ masses and muscle mass in relation to FFM, a general linear regression model was used with FFM as a dependent variable. Linear regression models examined the association between residuals of the FFM to REE relationship, and differences between REE and REEc, HOMA, CRP and thyroid hormones. A paired *t*-test was used to analyze the significance of the difference (Δ) between REE and REEc, and the differences were also plotted against age. A *p*-value < 0.05 was accepted as the limit of significance.

## 3. Results

### 3.1. Body Composition

Body composition characteristics of the study population are presented in Table 1. Men were heavier and taller than women, and they had higher BMI, FFM and organ masses. Visceral adipose tissue volumes (VAT) were higher in men, whereas total adipose tissue was higher in women. In both sexes, there were age-related decreases in the individual components of FFM. By contrast, in men, heart mass and VAT increased with age. Relative amounts of tissue and organ masses as part of FFM showed an age-related decrease of muscle and spleen mass in women and men. In contrast, masses of heart, brain, liver, kidneys as well as bone mineral in relation to FFM, increased during aging, which could be explained by co-occurring FFM decline. Compared to men, women had a lower body density but higher FFM hydration. In both sexes, body density decreased with increasing age; in contrast, FFM hydration increased in women only.

### 3.2. REE

There were significant age and sex effects on REE, REE_FFM_ and the difference between REE to REEc (ΔREE-REEc), as calculated using the age-unspecific algorithm of Elia *et al.*(Table 2; see Methods). In contrast, no sex and age-differences in the ΔREE-REEc were observed using the age-specific algorithms of Wang *et al.* (Table 2; see Methods). Compared with women, men had higher levels of free triiodothyronineand lower levels of thyroid-stimulating hormone as well as free thyroxin. In women, only free thyroxin levels showed an age-related increase. This was also true in men, whereas thyroid-stimulating hormone and free triiodothyroninedecreased with age in men.

**Figure 1:**
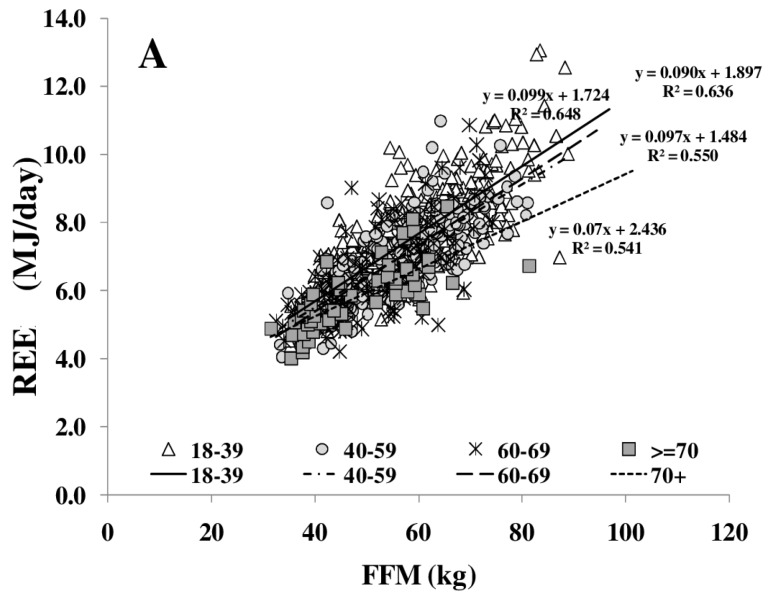

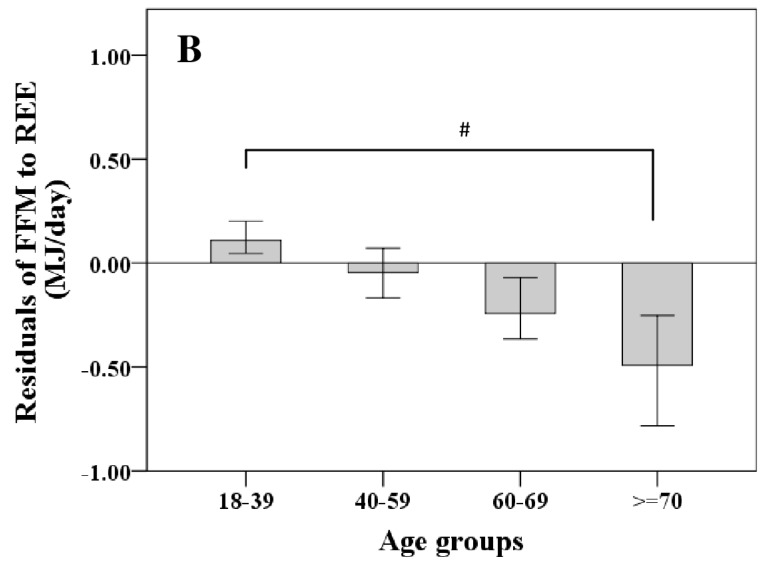
Age-dependent decrease in fat free mass (FFM)-resting energy expenditure (REE) relationship (**A**) and their residuals in different age groups (median; 95% CI) (**B**). Significant differences between age-groups are indicated by # as tested by Kruskal-Wallis-test (*n* = 714). FFM was assessed by Air Displacement Plethysmography (ADP) (for details, see Methods).

**Figure 2:**
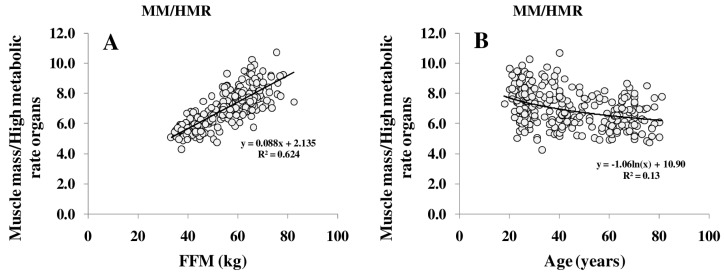
Relationship between FFM and the ratio of muscle mass (MM) to high metabolic rate organs (HMR) per kg (**A**) and between the MM/HMR-ratio and age (**B**). HMR is the sum of masses of brain, heart, liver and kidneys.FFM was assessed by ADP, organ masses were assessed by whole body Magnetic Resonance Imaging (MRI) (for details, see Methods) (*n* = 369).

REE increased with FFM (Figure 1A); however, REE related to FFM decreased with age. Comparing different age groups, residuals of the REE-FFM relationship turned from positive to negative residuals with increasing age (Figure 1B). The ratio of muscle mass (as major low metabolic rate tissue) to high metabolic rate organ masses (HMR), and thus the relative proportion of MM to HMR, increased with FFM (Figure 2A) and decreased nonlinearly with age (Figure 2B).

**Figure 3:**
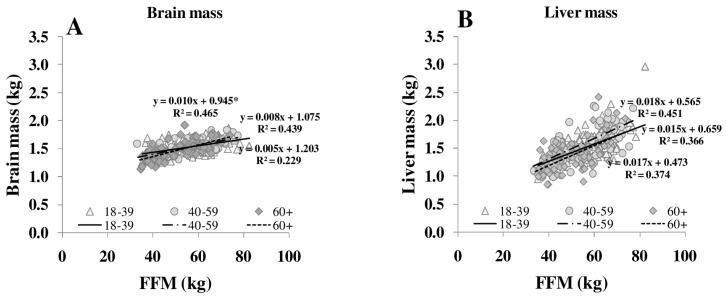

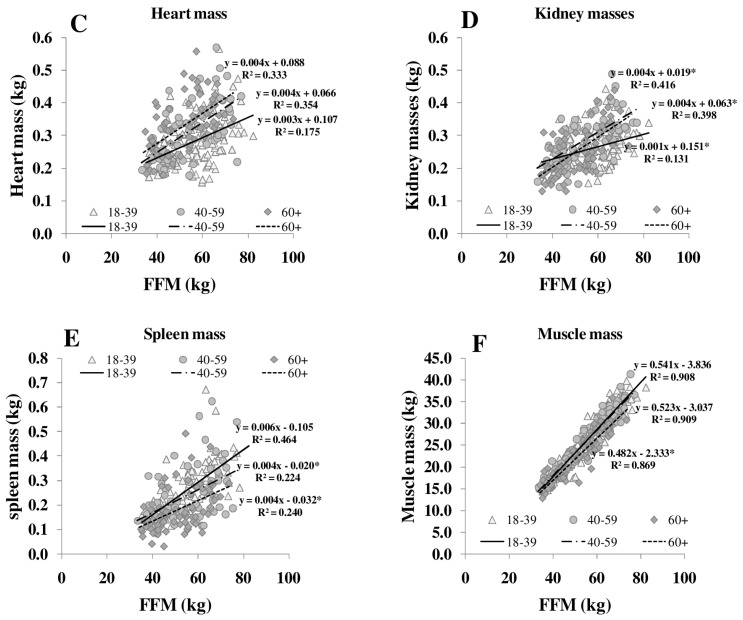
Relationships between FFM and masses of brain (**A**), liver (**B**), heart (**C**), kidneys (**D**), spleen (**E**) and skeletal muscle (**F**) in different age groups. FFM was assessed by ADP, organ masses were assessed by whole body MRI (*n* = 369). * significant difference between youngest and other age groups (*p* < 0.05).

The ratio of MM/FFM increased with increasing FFM, whereas the HMR/FFM ratio remained stable. The association between FFM, MM/FFM and HMR/FFM in young (18–39 years) middle-aged (40–59 years) and older (≥60 years) subjects showed no significant differences between the age groups. Comparing age groups, the relationship between FFM to MM/FFM became weaker (R^2^ between 0.11 and 0.06) but FFM to HMR/FFM relationship remained constant (R^2^ between 0.65 and 0.50). However, age affected the relationship of individual organ masses (*i.e.*, heart, spleen and skeletal muscle) to FFM (Figure 3).

**Figure 4:**
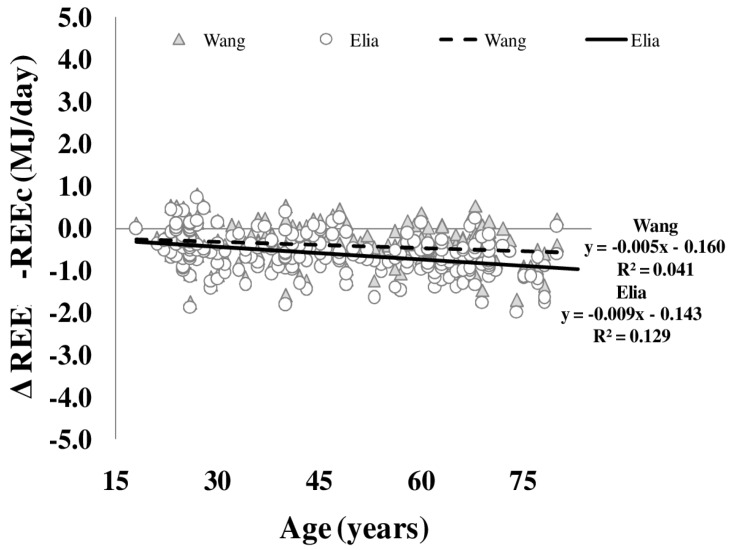
Age-dependency of differences between measured and calculated resting energy expenditure (REE–REEc). REEc was calculated from organ/tissue masses (as assessed by whole body MRI times organ- and tissue-mass-specific metabolic rates as published by Elia (**circles**) and Wang (**triangles**, *i.e.*, using age-adjusted specific metabolic rates) (*n* = 217) (for details, see Methods).

Differences between REE and REEc were plotted against age (Figure 4). Both REEc calculated according to either Elia or Wang *et al.* (see Methods) showed a significant bias of REE with age. This age bias was not explained by whole body density or FFM hydration. Plasma levels of free triiodothyronine explained 4.7% (Elia) and 2.5% (Wang *et al.*) of the variance in REE and REEc bias.

### 3.3. Cardiometabolic Risk

Table 3 shows cardiometabolic risk factors. Overall, there were no sex differences in biomarkers of inflammation and insulin resistance. Plasma levels of C-reactive protein significantly increased with age in women only. In contrast, an age-associated increase in HOMA index was only observed in men.

In multivariate regression analysis variance of REE-FFM, residuals were explained by HOMA (4.2%), CRP (2.0%) and TSH (1.4%). However, MM/FFM and HMR/FFM ratios accounted for 11.8% of REE-FFM residuals, whereas age added 1.9% to their variance.

## 4. Discussion

Both REE and the relationship between REE and FFM decreased with age [25]. The decrease in REE is explained by a reduction in FFM as well as by changes in the composition of FFM in normal and overweight subjects (Table 1, Figure 3). With increasing FFM, the proportion of HMR to FFM remained relatively constant, whereas the MM to FFM ratio decreased with age. Considering individual organ and tissue masses, heart mass and spleen masses per FFM increased with age (Figure 3). In a previous study of our group, Bosy-Westphal *et al.* [3] had already described that heart mass explained 58% of the variance in the difference between REE and REEc in elderly subjects. A higher heart mass with age was seen as a compensatory effect on chronic hypertensive load or hypertrophy of cardiocytes.

In addition to the age-related (i) decreases in FFM and (ii) changes in the proportion of organ/tissues mass to FFM, alterations of specific metabolic rates of individual organs and tissues add to age-related changes in REE. To address this issue, REE was calculated (REEc) based on organ and tissue masses times specific tissue metabolic rates. Elia [27] was first to publish metabolic rates of individual organs and tissues assuming constant values across lifespan. By contrast, Gallagher *et al.* [4] showed that specific metabolic rates based on data of younger subjects do not resemble the respective metabolic rates in elderly subjects. In a previous work, Wang *et al.* [13] calculated that specific metabolic rates of organs and tissues changed with age resulting in corrections of Elias constants. Anyhow, the present data revealed that the differences between REE and REEc increased with age. This was obvious for both the Elia and Wang predictions (Figure 4). The bias between REE and REEc could not be explained by the age-related changes in whole body density or FFM hydration. In our opinion, this supports the idea that the physical property of organ and tissue masses are not related to age-related changes in REE.

In the Baltimore Longitudinal Study of Aging, Ruggiero *et al.* [14] and Fabbri *et al.* [16] have already shown that higher resting metabolic rates in age were associated with multi-morbidity and mortality. We now add the finding that age-related changes in the REE-FFM-association (*i.e.*, the REE-FFMresiduals) were related to inflammation. Thus, the variance in the FFM to REE relationship could reflect health status.

A limitation of this study was the assumption of age-independent constant organ and tissue densities that affects the estimate of organ and tissue masses from their volumes. In addition, we have used cross-sectional data only; thus, our data cannot give a future prospect on individual aging. The fact that data analysis included normal and overweight subjects (BMI < 30 kg/m^2^) only could be seen as critical, but this cut off was chosen to cover current normal BMI ranges for all young as well as older adults.

## 5. Conclusions

In conclusion, age-related changes of REE relate to decreases in FFM as well as alterations in the REE-FFM relationship. In addition, proportional changes in FFM composition (*i.e.*, the organ and tissue mass ratios to FFM) plus decreases in specific metabolic rates of organs and tissues add to the decrease in REE with age. The variance in the REE-FFM association is related to inflammation.

## Figures and Tables

**Table 1:** Physical characteristics of the main study population (*n* = 714) and subgroup with detailed body composition (*n* = 369).

Characteristic Title	Women				Men					
	All	18–39 Years	40–59 Years	60–69 Years	70+ Years	All	18–39 Years	40–59 Years	60–69 Years	70+ Years
*Number of subjects*	*346*	*162*	*103*	*59*	*22*	*368*	*166*	*119*	*60*	*23*
Weight (kg)	67.1 [60.3–72.8] *	66.2 [59.1–731]	68.3 [62.9–74.6]	68.8 [62.6–72.7]	61.7 [57.9–70.6]	81.1 [73.3–88.6]	79.1 [72.5–88.6]	83.2 [76.8–90.1]	79.6 [74.0–87.6]	75.9 [71.5–83.6] #
Height (m)	1.66 [1.62–1.72] *	1.70 [1.64–1.73]	1.66 [1.62–1.71]	1.64 [1.60–1.67]	1.61 [1.57–1.64] #	1.79 [1.76–1.84]	1.80 [1.77–1.86]	1.81 [1.76–1.84]	1.76 [1.72–1.79]	1.74 [1.72–1.77] #
BMI (kg/m^2^)	24.1 [21.7–26.7] *	22.9 [21.1–25.6]	24.8 [22.8–26.9]	25.4 [23.3–27.3]	23.9 [21.5–27.7] #	25.0 [22.9–27.5]	23.6 [22.2–26.8]	25.6 [23.6–27.5]	26.1 [24.9–28.4]	24.6 [23.7–27.8] #
FFM_ADP_(kg)	44.6 [40.7–47.9] *	46.1 [42.7–50.1]	44.9 [41.5–49.2]	42.2 [37.8–45.2]	38.8 [37.4–41.9] #	62.5 [57.9–67.5]	64.8 [59.4–69.4]	63.0 [59.4–67.5]	58.9 [54.8–64.3]	57.1 [54.1–59.4] #
FM_ADP_ (kg)	22.4 [17.5–27.5] *	20.3 [16.2–25.4]	23.0 [17.8–23.3]	26.3 [22.0–31.0]	22.2 [18.6–30.5] #	18.5 [12.9–23.1]	14.2 [10.0–21.4]	20.5 [15.4–25.0]	20.8 [18.1–24.8]	18.1 [15.4–27.5] #
Body density (kg/L)	1.02 [1.01–1.03] *	1.03 [1.02–1.03]	1.02 [1.01–1.03]	1.01 [1.00–1.02]	1.01 [1.00–1.03] #	1.04 [1.03–1.06]	1.05 [1.04–1.07]	1.04 [1.03–1.05]	1.04 [1.03–1.05]	1.04 [1.03–1.05] #
FFM hydration (L/kg) (*n* = 290)	0.75 [0.73–0.78] *	0.74 [0.71–0.76]	0.75 [0.73–0.78]	0.77 [0.72–0.79]	0.79 [0.76–0.79] #	0.73 [0.70–0.76]	0.73 [0.70–0.76]	0.73 [0.70–0.76]	0.74 [0.71–0.76]	0.74 [0.71–0.74]
*Detailed body composition*										
Muscle mass_MRI_ (kg) (*n* = 369)	19.6 [17.5–21.8] *	20.7 [18.7–23.3]	20.3 [18.5–22.3]	17.5 [16.4–19.3]	16.7 [15.2–17.5] #	29.7 [26.9–32.8]	31.7 [18.7–23.3]	30.6 [27.8–32.3]	27.1 [24.4–29.6]	25.6 [22.2–27.1] #
*% Muscle mass_MRI_*	*44.4* [*42.4–46.7*] *	*45.3* [*43.1–47.7*]	*44.5* [*43.5–47.0*]	*42.7* [*41.3–45.5*]	*39.9* [*38.7–41.3*] *#*	*47.4* [*45.4–49.8*]	*48.4* [*46.2–50.6*] *#*	*47.4* [*45.8–50.4*]	*45.8* [*42.8–47.2*]	*44.8* [*40.8–46.1*] *#*
Brain mass_MRI_ (kg) (*n* = 266)	1.41 [1.35–1.48] *	1.45 [1.39–1.51]	1.44 [1.36–1.48]	1.38 [1.35–1.41]	1.25 [1.23–1.41] #	1.57 [1.49–1.67]	1.56 [1.49–1.67]	1.65 [1.55–1.70]	1.56 [1.47–1.63]	1.54 [1.43–1.61]
*% Brain mass_MRI_*	*3.20* [*2.91–3.42*] *	*3.10* [*2.84–3.39*]	*3.15* [*2.85–3.44*]	*3.26* [*3.13–3.53*]	*3.25* [*3.05–3.61*] *#*	*2.52* [*2.33–2.72*]	*2.45* [*2.30–2.67*]	*2.40* [*2.30–2.65*]	*2.62* [*2.43–2.75*]	*2.75* [*2.41–2.98*] *#*
Heart mass_MRI_ (kg) (*n* = 260)	0.25 [0.21–0.32] *	0.24 [0.22–0.29]	0.25 [0.22–0.33]	0.26 [0.23–0.33]	0.21 [0.18–0.28] #	0.32 [0.27–0.39]	0.29 [0.25–0.33]	0.33 [0.29–0.38]	0.34 [0.29–0.39]	0.39 [0.29–0.46] #
*% Heart mass_MRI_*	*0.58* [*0.49–0.69*] *	*0.49* [*0.44–0.60*]	*0.58* [*0.48–0.68*]	*0.71* [*0.55–0.77*]	*0.60* [*0.51–0.70*] *#*	*0.51* [*0.44–0.61*]	*0.46* [*0.38–0.58*]	*0.54* [*0.46–0.61*]	*0.53* [*0.49–0.63*]	*0.66* [*0.58–0.81*] *#*
Liver mass_MRI_ (kg) (*n* = 266)	1.36 [1.18–1.52] *	1.42 [1.28–1.56]	1.39 [1.23–1.54]	1.14 [1.03–1.41]	1.24 [1.02–1.37] #	1.57 [1.39–1.81]	1.57 [1.40–1.71]	1.79 [1.49–1.97]	1.54 [1.39–1.68]	1.33 [1.22–1.49] #
*% Liver mass_MRI_*	*3.01* [*2.76–3.30*] *	*3.01* [*2.79–3.25*]	*3.04* [*2.77–3.43*]	*2.8* [*2.64–3.37*]	*3.10* [*2.68–3.36*]	*2.55* [*2.27–2.78*]	*2.47* [*2.24–2.69*]	*2.72* [*2.41–2.92*]	*2.60* [*2.38–2.84*]	*2.34* [*2.17–2.71*] *#*
Kidney masses_MRI_ (kg) (*n* = 265)	0.22 [0.19–0.27] *	0.24 [0.21–0.28]	0.26 [0.21–0.28]	0.19 [0.18–0.27]	0.19 [0.16–0.21] #	0.28 [0.23–0.33]	0.26 [0.23–0.31]	0.32 [0.28–0.33]	0.31 [0.25–0.37]	0.23 [0.21–0.29] #
*% Kidney masses_MRI_*	*0.51* [*0.44–0.61*] *	*0.50* [*0.45–0.58*]	*0.53* [*0.45–0.64*]	*0.51* [*0.45–0.63*]	*0.42* [*0.39–0.54*]	*0.45* [*0.39–0.53*]	*0.40* [*0.35–0.48*]	*0.50* [*0.44–0.57*]	*0.53* [*0.45–0.59*]	*0.40* [*0.38–0.50*] *#*
Spleen mass (kg) (*n* = 229)	0.17 [0.14–0.21] *	0.18 [0.16–0.24]	0.18 [0.15–0.21]	0.14 [0.10–0.17]	0.12 [0.09–0.17] #	0.27 [0.18–0.35]	0.32 [0.24–0.39]	0.28 [0.18–0.36]	0.23 [0.16–0.28]	0.15 [0.12–0.27] #
*% Spleen mass_MRI_*	*0.37* [*0.32–0.47*]	*0.41* [*0.34–0.48*]	*0.37* [*0.33–0.50*]	*0.34* [*0.27–0.38*]	*0.29* [*0.24–0.42*] *#*	*0.40* [*0.28–0.56*] *	*0.51* [*0.38–0.58*]	*0.40* [*0.25–0.55*]	*0.35* [*0.27–0.43*]	*0.26* [*0.21–0.43*] *#*
Residual mass_MRI_ (kg) (*n* = 224)	17.1 [14.3–19.8] *	17.5 [15.9–20.4]	18.9 [15.9–20.7]	18.1 [15.6–24.9]	27.0 [23.8–28.7] #	22.8 [19.6–27.1]	22.7 [19.9–25.6]	24.7 [22.1–27.9]	24.2 [19.9–28.1]	39.0 [34.9–40.9] #
*% Residual mass_MRI_*	*37.1* [*32.9–42.9*]	*33.7* [*29.4–37.3*]	*36.4* [*32.6–39.7*]	*41.6* [*37.2–61.8*]	*66.6* [*60.4–68.9*] *#*	*35.6* [*31.9–39.8*]	*31.4* [*27.9–35.9*]	*35.2* [*32.5–37.8*]	*38.1* [*33.2–43.8*]	*66.9* [*48.8–71.1*] *#*
Muscle mass/ Organ mass (*n* = 260)	5.9 [5.5–6.4] *	6.1 [5.6–6.7]	6.2 [5.7–6.6]	5.6 [5.4–5.9]	5.6 [5.1–6.1] #	7.7 [7.0–8.3]	8.2 [7.6–8.8]	7.4 [6.8–8.0]	6.9 [6.6–7.5]	6.9 [6.6–7.7] #
Adipose tissue_MRI_ (L) (*n* = 369)	23.5 [18.9–29.4] *	22.9 [18.4–28.6]	25.0 [20.5–33.7]	23.9 [18.6–28.6]	22.7 [17.8–28.5] #	19.03 [14.4–24.1]	15.8 [11.9–22.4]	21.7 [18.1–25.9]	20.9 [18.9–25.9]	16.3 [14.1–20.3] #
Visceral adipose tissue_MRI_ (L) (*n* = 369)	1.24 [0.63–2.08]	0.87 [0.45–1.43]	1.33 [0.82–2.44]	1.65 [1.12–2.54]	1.95 [1.24–2.61] #	2.71 [1.43–4.17]	1.71 [0.84–3.35]	2.96 [1.85–4.66]	4.18 [3.74–6.08]	3.11 [2.14–5.43] #
Bone mineral_DXA_ (kg) (*n* = 329)	4.2 [3.7–4.7] *	4.4 [3.9–4.7]	4.2 [3.7–4.6]	3.8 [3.6–4.7]	5.5 [3.8–6.4] #	5.4 [4.8–5.9]	5.4 [4.8–5.7]	5.1 [4.8–5.7]	5.6 [4.8–6.7]	6.7 [5.5–7.5] #
*%Bone mineral_DXA_*	*9.3* [*8.7–10.0*] *	*9.3* [*8.7–9.9*]	*9.2* [*8.6–9.8*]	*9.1* [*8.3–10.9*]	*13.1* [*10.1–14.7*] *#*	*8.4* [*7.9–8.9*]	*8.2* [*7.7–8.7*]	*8.1* [*7.8–8.5*]	*8.8* [*8.5–10.1*]	*11.7* [*9.7–12.9*] *#*

BMI: body mass index, FM: fat mass, FFM: fat free mass. * significant difference between women and men (Mann-Whitney U-test); # significant difference between age-groups (Kruskal-Wallis-test) all data median (Interquartil Range).

**Table 2:** Measured REEm and calculated REEc resting energy expenditure and differences between measured and calculated energy expenditure for the study population (*n* = 714).

Characteristic Title	Women					Men				
	All	18–39 Years	40–59 Years	60–69 Years	70+ Years	All	18–39 Years	40–59 Years	60–69 Years	70+ Years
*Number of subjects*	*346*	*162*	*103*	*59*	*22*	*368*	*166*	*119*	*60*	*23*
***Resting energy expenditure***										
REE (MJ/day) (*n* = 714)	5.8 [5.3–6.4] *	6.1 [5.6–6.6]	5.8 [5.4–6.4]	5.5 [4.9–5.9]	5.0 [4.7–5.3] #	7.5 [6.8–8.3]	7.8 [7.2–8.6] #	7.7 [6.9–8.3]	6.9 [6.1–7.8]	6.3 [5.9–6.7]
REE_FFM_ (MJ/day) (*n* = 714)	5.8 [4.9–6.6] *	6.2 [5.4–6.9]	5.9 [4.9–6.8]	5.3 [4.5–6.5]	4.7 [3.9–5.1] #	7.5 [6.2–8.8]	7.9 [6.7–9.4] #	7.7 [6.4–8.5]	6.4 [5.5–7.8]	5.7 [4.8–6.9]
REEc by Elia (MJ/day) (*n* = 217)	5.9 [5.6–6.5] *	6.0 [5.7–6.7]	6.1 [5.7–6.6]	5.7 [5.5–6.1]	5.8 [5.4–6.0]	7.6 [6.9–8.0]	7.5 [6.9–7.9]	7.9 [7.3–8.3]	7.7 [6.7–8.0]	7.5 [6.9–7.9]
ΔREE–REEc by Elia (MJ/day) (*n* = 217)	−0.56 [−0.78–0.29] *	−0.42 [−0.67–0.19]	−0.66 [−0.77–0.16]	−0.66 [−0.92–0.44]	−0.84 [−1.01–0.59] #	−0.63 [−1.04–0.37]	−0.48 [−0.88–0.03] #	−0.62 [−0.96–0.29]	−0.79 [−1.22–0.56]	−1.03 [−1.39–0.39]
REEc by Wang *et al.* (MJ/day) (*n* = 217)	5.8 [5.4–6.3] *	5.9 [5.4–6.3]	5.9 [5.5–6.4]	5.5 [5.2–5.8]	5.6 [5.1–5.8] #	7.4 [6.9–7.8]	7.4 [6.8–7.8]	7.7 [7.1–7.9]	7.3 [6.4–7.7]	7.2 [6.6–7.6]
ΔREE–REEc by Wang *et al.* (MJ/day) (*n* = 217)	−0.41 [−0.60–0.19]	−0.31 [−0.57–0.08]	−0.46 [−0.61–0.08]	−0.41 [−0.61–0.22]	−0.60 [−0.81–0.41]	−0.41 [−0.81–0.07]	−0.33 [−0.73–0.09]	−0.38 [−0.75–0.01]	−0.46 [−0.82–0.20]	−0.78 [−1.12–0.08]
*Energy metabolism related hormones*										
Thyroid-stimulating hormone (mU/L) (*n* = 566)	1.70 [1.16–2.49] *	1.92 [1.31–2.76]	1.43 [1.09–2.35]	1.69 [1.05–2.69]	1.46 [1.09–1.60]	1.49 [1.04–2.25]	1.83 [1.20-2.64]	1.33 [0.94–1.85]	1.39 [1.04-2.06]	1.44 [1.04–2.06] #
Free triiodothryonin (pmol/L) (*n* = 566)	3.63 [3.12–4.25] *	3.69 [3.18–4.34]	3.64 [3.04-4.22]	3.46 [2.97–3.91]	3.49 [2.83–4.13]	3.90 [3.26–4.59]	3.92 [3.20-4.67]	4.01 [3.42–4.64]	3.52 [3.19–4.04]	3.60 [2.75-4.08] #
Free thyroxin (pmol/L) (*n* = 566)	18.79 [12.19-15.82] *	13.21 [12.09–14.95]	14.30 [12.16-16.27]	14.21 [12.38–19.37]	17.41 [16.44–19.37] #	14.52 [12.29–16.68]	13.64 [10.50–16.04]	15.40 [13.84–17.33]	14.47 [12.21–17.46]	16.16 [15.16–17.69] #

REE: resting energy expenditure, REE_FFM_: fat free mass adjusted resting energy expenditure, REEc: calculated resting energy expenditure, ΔREE–REEc: difference between REE and REEc; * significant difference between women and men (Mann-Whitney U-test); # significant difference between age-groups (Kruskal-Wallis-test) all data median (Interquartil Range).

**Table 3:** Characteristics of metabolic risk factors of the study population (*n* = 714).

Characteristic Title	Women					Men				
	All	18–39 Years	40–59 Years	60–69 Years	70+ Years	All	18–39 Years	40–59 Years	60–69 Years	70+ Years
*Number of subjects*	*346*	*162*	*103*	*59*	*22*	*368*	*166*	*119*	*60*	*23*
*Inflammation*										
CRP (mg/l) (*n* = 437)	0.69 [0.20–1.75]	0.92 [0.35–2.36]	0.81 [0.25–1.53]	0.41 [0.17–1.79]	0.20 [0.10–0.35] #	0.43 [0.16–1.29]	0.32 [0.14–1.23]	0.70 [0.28–1.67]	0.63 [0.23–1.19]	0.20 [0.10–0.78]
*Insulin resistance*										
Glucose (mg/dl) (*n* = 551)	90.6 [84.8–96.6]	87.9 [83.9–92.4]	91.6 [84.5–97.0]	96.0 [92.0–103.0]	93.5 [85.5–105.5]	94.7 [87.4–101.9]	90.0 [62.4–96.7]	97.0 [91.9–104.6]	99.5 [92.1–108.0]	96.0 [89.8–102.5]
Insulin (mU/dl) (*n* = 530)	8.8 [6.6–12.3]	9.2 [6.9–12.8]	7.8 [5.9–11.4]	9.5 [6.9–12.5]	8.9 [6.0–11.5]	8.1 [6.3–10.9]	8.2 [6.0–10.4]	8.2 [6.4–12.1]	7.8 [6.6–11.2]	7.5 [5.9–11.3]
HOMA (*n* = 529)	1.93 [1.38–2.80]	2.06 [1.55–3.07]	1.78 [1.09–2.59]	2.04 [1.56–2.76]	1.85 [1.29–2.81]	1.73 [1.38–2.37]	1.59 [1.19–2.16]	1.95 [1.42–2.37]	2.09 [1.66–3.31]	1.91 [1.49–2.51] #

# significant difference between age-groups (Kruskal-Wallis-test) all data median (Interquartil Range).

## Abbreviations

The following abbreviations are used in this manuscript:

BMCDXA	bone mineral content measured by dual energy X-ray absorptiometry
DXA	dual energy X-ray absorptiometry
FFM	fat free mass by DXA
FM	fat mass
MJ/day	mega joule per day
MM	skeletal muscle mass
MRI	magnetic resonance imaging
HMR	high metabolic rate organs
REE	measured resting energy expenditure
REEc	REE calculated from organ/tissue masses times their specific metabolic rates
∆ REE-REEc	difference between measured resting energy expenditure and calculated resting energy expenditure
